# Multidrug-resistant mammary pathogenic *Escherichia coli* ST479 isolated from Holstein dairy cows in Jiangsu, China

**DOI:** 10.3389/fmicb.2026.1737656

**Published:** 2026-03-03

**Authors:** Kun Wang, Minghui Zhang, Yujie Li, Mingxun Li, Yujia Sun, Zhangping Yang

**Affiliations:** 1 College of Animal Science and Technology, Yangzhou University, Yangzhou, China; 2 Joint International Research Laboratory of Agriculture and Agri-Product Safety Ministry of Education, Yangzhou University, Yangzhou, China

**Keywords:** bovine mastitis, *Escherichia coli* ST479, MLST, multidrug resistance, ST479

## Abstract

Mastitis is one of the most prevalent diseases in dairy cattle farming, causing significant economic losses to the industry. *Escherichia coli* (*E. coli*) is a major infectious pathogen responsible for bovine mastitis. This study conducted molecular and phenotypic characterization analyses of *E. coli* strains isolated from milk samples of clinical mastitis cases in Jiangsu Province, China. Through antibiotic susceptibility testing, multilocus sequence typing (MLST), whole-genome sequencing analysis, and comparative assessment of mammary pathogenicity in cellular and murine models. The results demonstrate that *E. coli* ST479 is a multidrug-resistant mastitis-causing strain capable of producing extended-spectrum β-lactamases (ESBLs) and exhibits a globally widespread distribution. In experimental models, ST479 invaded bovine mammary epithelial cells and induced mastitis in mice. Notably, it elicited a moderated inflammatory response compared to the reference strain ATCC 25922, suggesting a distinct host-pathogen interaction profile The emergence of multidrug-resistant clonal strains such as ST479, capable of causing mastitis and producing ESBLs, represents a concern for dairy farm health management due to its potential to limit treatment options. Given the detection of ST479 in multiple regions around the world and its multiple antibiotic drug resistance, we emphasize the need for enhanced surveillance and management of such strains in dairy farms to safeguard animal health and ensure sustainable production.

## 1 Introduction

Mastitis is a common disease in dairy cows that leads to decreased milk production and affects milk quality, resulting in significant economic losses for related industries. It results in nearly $2 billion in annual losses for the U.S. livestock industry and poses a risk to animal health (Zigo et al., 2021). One of the primary bacterial agents causing mastitis in dairy cows is *Escherichia coli* (*E. coli*), which is a zoonotic pathogen. Despite belonging to the same genus, studies have shown that mastitis-causing *E. coli* strains have distinct genomic characteristics (Kusza and Bagi, 2025). Environmental factors significantly influence the evolution and persistence of pathogens, necessitating that treatment strategies account for local strain variations and resistance patterns. Additionally, population genetic studies indicate that the bacterial genotype often corresponds to the host species specificity (Maier et al., 2024). Consequently, a growing number of researchers are inclined to classify mastitis-associated *E. coli* as a distinct group termed Mastitis Pathogenic *E. coli* (MPEC) (Goulart and Mellata, 2022). Although the concept of MPEC has been proposed for some time, the defining characteristics of this group have yet to be fully established. Recent genomic comparative studies have not consistently identified genes that are more prevalent in mastitis-associated strains compared to non-pathogenic strains (Blum et al., 2018; Sun et al., 2021). In addition, although a variety of PFGE genotypes may be found among MPEC, mastitis pathogenic strains were less genotypically diverse than environmental strains, as shown by multi-locus sequence typing (Blum et al., 2015). Furthermore, the relationship between virulence-associated genes and different hosts or colonization sites remains unclear. Therefore, more detailed investigations into mastitis-associated *E. coli* are necessary to determine the epidemiological characteristics of MPEC and the genes required for colonization and transmission.

Multi-locus sequence typing (MLST) is regarded as one of the effective tools for studying the prevalence and genetic diversity of pathogens in a specific region (Maiden et al., 2013). This method facilitates the precise characterization of pathogenic strains, making it essential for research in epidemiology, population dynamics, and evolutionary biology. Researchers have analyzed tropical Candida isolates from Italy and Africa using MLST, discovering numerous new genotypes and a high level of genetic diversity (Dougue et al., 2022). A study from Germany found that *E. coli* isolates from livestock and food predominantly belonged to three sequence types (ST648, ST410 and ST167). Furthermore, the bla_*CTX*–*M*–15_ gene was identified in multidrug-resistant *E. coli* clones with pathogenic potential (Fischer et al., 2014). Clinical bacterial isolates have demonstrated an association between prevalent genotypes, specific virulence traits, and geographical distribution. The increasing level of population mobility not only shapes the unique characteristics of the prevalence and transmission of bacterial antibiotic resistance but also indirectly alters the global patterns of its dissemination (Luo et al., 2024). However, there is still a lack of detailed MLST evaluations and related genetic studies on pathogens in farms, particularly concerning MPEC.

Surveillance and mechanistic studies of bacterial drug resistance represent the most critical measures for controlling the spread of resistant bacteria (Tacconelli et al., 2018). In dairy cattle husbandry, the emergence and dissemination of multidrug-resistant mastitis pathogens pose a significant challenge to disease management. In China’s rapidly expanding dairy sector, insufficient regulation of antimicrobial use and rising antimicrobial resistance are increasingly prominent. According to statistics, in 2013, 52% of antimicrobials in China were used for animal diseases (Xiong et al., 2024). The extensive use of various antibiotics in livestock farming has been associated with significant shifts in the resistance patterns of mastitis pathogens in dairy cows, posing growing challenges for both prevention and treatment (Velasco Garcia et al., 2025). The emergence of extended-spectrum β-lactamases (ESBLs) and plasmid-encoded AmpC enzymes has rendered many commonly used antibiotics ineffective (Dolejska and Papagiannitsis, 2018). In addition to creating resistance, these antimicrobial agents also affect microbial communities, leading to changes in the genetic diversity of mastitis pathogens (Park et al., 2012). In livestock and poultry populations, studies have indicated that the use of specific antimicrobial agents may be associated with increased levels of antimicrobial resistance in *E. coli* isolated from pigs, poultry, and cattle (Yang et al., 2022). Research findings from various countries and regions indicate that specific sequence types of bacteria have both a transmission advantage and increased pathogenicity in livestock, often carrying identical antibiotic-resistance genes (Aslam et al., 2021). Therefore, to mitigate the escalating threat of antimicrobial resistance, it is imperative to implement strict policies that minimize the non-therapeutic use of antimicrobial agents in livestock farming, reserving their application primarily for therapeutic purposes under veterinary oversight.

This study aims to characterize the genotypes, antibiotic resistance profiles, virulence factors features of *E. coli* isolates obtained from clinical bovine mastitis milk samples in Jiangsu Province, China. In addition, we compared the responses of the hosts (cells and murine models) to ST479 with their responses to the reference strain ATCC 25922. Our research findings indicate that ST479 produces ESBLs and carries the bla_*CTX*–*M*–55_ gene. In vitro and in vivo models confirmed its ability to invade mammary epithelial cells and induce inflammatory pathology. A noteworthy finding was the differential host response elicited by ST479 compared to the reference strain, providing new clues about its interaction with the mammary gland. Our research findings indicate that the strain of *E. coli*, identified as ST479, produces ESBLs and carries the bla_*CTX*–*M*–55_ gene. ST479 has been identified in multiple countries and regions worldwide and is found in various animal hosts, including cattle and pigs. This study provides new insights into the molecular and pathogenic characteristics of a multidrug-resistant *E. coli* ST479 clone associated with bovine mastitis, offering a reference for related research.

## 2 Materials and methods

### 2.1 Materials

The antibiotic susceptibility testing strips were purchased from Hangwei Company (Hangzhou, China). The *E. coli* ATCC 25922, was obtained from the American Type Culture Collection. The primary antibodies, including phosphorylated NF-κB p65, Phospho-NF-κB p65, NLRP6, TLR4, Occludin, ZO-1, and β-actin, were all sourced from Proteintech (Wuhan, China). Goat anti-rabbit or rabbit anti-mouse secondary antibodies were acquired from Beyotime (Shanghai, China). The cell viability assay kit was also procured from Beyotime (Shanghai, China).

All the experimental methods in this study were evaluated and approved by the Experimental Animal Ethics Committee of Yangzhou University (NO. 202503247). The environmental conditions of this facility meet the requirements for standard animal experimentation facilities outlined in the Chinese National Standard Experimental Animal Environment and Facilities (GB14925–2023). Animal husbandry management and experimental procedures are conducted in compliance with all applicable Chinese regulations.

### 2.2 Sample collection

From June to September 2024, this study collected milk samples from dairy cows with clinical mastitis across four intensive dairy farms (herd size > 1,000 cows each) in Xuzhou, Suqian, Huaian, and Yancheng cities, Jiangsu Province, China. A total of 96 samples were obtained based on the availability of eligible cases during the study period (convenience sampling). Clinical mastitis was identified and classified based on a modified version of the National Mastitis Council (NMC) scoring system. Only cases presenting with both local signs of udder inflammation (redness, swelling, heat, or pain) and visible abnormalities in the milk (e.g., flakes, clots, or watery secretion) were included (i.e., bovine individuals with a clinical severity score of 2). To objectively confirm the presence of mammary gland inflammation and to standardize the inclusion threshold across all participating farms, a positive California Mastitis Test (CMT) result ( ≥ ++) was required. The procedures for sample collection and processing are as follows.

(a) Aseptic sampling: Prior to collection, the teat end was thoroughly disinfected with 70% ethanol and allowed to dry completely. After forestripping (discarding the first three streams of milk), samples were collected aseptically into sterile 50 mL polypropylene tubes without contacting the teat or surrounding tissues, and transported promptly to the laboratory.
(b) Primary isolation and contamination assessment (NMC guidelines): Upon arrival, each undiluted milk sample was streaked onto sheep blood agar plates using a sterile inoculating loop in a four-quadrant pattern. Plates were incubated aerobically at 37 °C for 24–48 h. Contamination definition: Any primary culture plate exhibiting more than two distinct colony morphologies was classified as contaminated and the entire sample was excluded from further analysis. Infection definition: If one or two colony morphologies were observed, the sample was considered non-contaminated. In such cases, the morphological characteristics (size, color, hemolysis, etc.) of the dominant colony (the colony type comprising > 80 % of the growth) were recorded, and this colony was operationally defined as the putative causative agent.
(c) Parallel dilution for clonal isolation (for sequencing and MLST): Immediately after the undiluted diagnostic culture was set up, and only for samples that had passed the initial contamination screen, a parallel series of ten-fold dilutions of the milk sample was prepared in sterile phosphate-buffered saline. An appropriate dilution (typically yielding 20–200 discrete colonies per plate) was selected, and 100 μL was spread onto a fresh sheep blood agar plate and incubated at 37 °C for 24 h. Colony morphologies on the dilution plate were compared with those on the undiluted diagnostic plate. From the dilution plate, a single, well-isolated colony that exactly matched the dominant colony morphology recorded in step (b) was picked with a sterile inoculating loop and streaked onto a new sheep blood agar plate for purification and biomass expansion (37 °C, 24 h). This procedure ensured the acquisition of genetically homogeneous clones, a prerequisite for high-quality DNA extraction for MLST and whole-genome sequencing (WGS). A single colony from the purified blood plate was then streaked onto eosin methylene blue (EMB) agar and incubated at 37 °C for 16–24 h. A single, well-isolated colony exhibiting the characteristic metallic sheen (phenotypic confirmation of *E. coli*) was inoculated into LB broth. Working strains were preserved in LB broth containing 25 % glycerol at –80 °C.

Of the 96 initial milk samples, 24 *E. coli* isolates were recovered following the exclusion of contaminated samples and the subculture procedure described above. All *E. coli* isolates characterized in this study originated from samples in which *E. coli* was the dominant species and were selected and purified through this entire workflow.

### 2.3 Bacterial species identification

Genomic DNA was extracted from the isolated *E. coli* strains using the Bacterial Genomic DNA Extraction Kit (DP302-02) (Tiangen, Beijing, China), following the manufacturer’s protocol. The procedure was briefly summarized as follows: Bacterial cultures grown overnight were collected by centrifugation, and the resulting cell pellets were resuspended in buffer. Cell lysis was achieved through lysozyme treatment, followed by protein digestion with Proteinase K. DNA was then purified using spin columns. The purified DNA was dissolved in elution buffer and stored at -20°C for subsequent PCR amplification and sequencing analysis. PCR amplification was performed using the extracted bacterial DNA as the template. The primer sequences for 16S rDNA identification are listed in Supplementary File 1. The PCR reaction mixture (total volume: 50 μL) consisted of 22.5 μL of 2 × Taq Master Mix, 2 μL each of forward and reverse primers, 1 μL of DNA template, and 22.5 μL of ultrapure water. The amplification program was set as follows: initial denaturation at 98°C for 2 min; followed by 30 cycles of denaturation at 98°C for 10 s, annealing at 58°C for 30 s, and extension at 72°C for 40 s; with a final extension at 72°C for 1 min. The PCR products were verified by 1% agarose gel electrophoresis and subsequently sent to Nanjing TsingKe Biotech Co., Ltd. for sequencing. The obtained sequences were subjected to BLAST analysis via the NCBI database^1^ to determine the genus of the pathogenic bacteria. And the automated microbial identification system (BioReader5000, France) was employed to differentiate between *E. coli* and Shigella spp. All isolates identified as *E. coli* were stored at -80°C.

### 2.4 Antimicrobial susceptibility testing

The agar disk diffusion method was employed to assess the antibiotic susceptibility of *E. coli* in Mueller-Hinton agar, as well as to detect the production of ESBLs. The following 18 antibiotic discs (Hangwei, China) were used: cefalexin (CA, 30 μg); ampicillin (AM, 10 μg); cephalothin (CF, 30 μg); ceftazidime (CAZ, 30μg); cefotaxime (CTX, 30 μg); cefepime (FEP, 30 μg); amoxicillin (AMC, 20 μg); meropenem (MEM, 10 μg); penicillin (P, 10U); Amikacin (AK, 30 μg); streptomycin (S, 10 μg); kanamycin (KAN, 30 μg); gentamicin (GM, 10 μg); tetracycline (TE, 30 μg); ciprofloxacin (CIP, 5 μg); chloramphenicol (C, 30 μg); trimethoprim-sulfamethoxazole (SXT, 20 μg); polymyxin B (PB, 300IU). The determination of drug susceptibility was based on interpretive criteria (inhibition zone diameters and MIC breakpoints for Enterobacteriaceae) published in the Clinical and Laboratory Standards Institute (CLSI) document M100 (32nd Edition), which provides standards for testing bacteria of human origin (Schuetz et al., 2025). *E. coli* ATCC25922 was employed as a quality control strain. In the absence of explicit standards for detecting bovine-origin *E. coli*, the aforementioned identification criteria represent a common and accepted practice in veterinary microbiology research. Using a sterile bacteriological swab, evenly streak the actively growing bacterial suspension across 100 mm diameter LB agar plates. Place 4–5 antibiotic sensitivity discs on each plate, ensuring a minimum center-to-center distance of 24 mm between adjacent discs to prevent overlap of inhibition zones and thereby avoid compromising measurement accuracy. The specific criteria for assessing bacterial susceptibility to antibiotics are provided in Table 1. Multidrug resistance was defined as resistance to at least one agent in three or more antimicrobial categories. The production of ESBLs was confirmed by resistance to third-generation cephalosporins and a difference of ≥ 5 mm in the zone of inhibition in the combination disk diffusion test (Magiorakos et al., 2012).

**TABLE 1:** *Escherichia coli* antimicrobial susceptibility criteria (disk diffusion method).

Antimicrobial agents (disk content)	Inhibition zone diameter breakpoint (mm)		
	Resistant (R)	Intermediate (I)	Susceptible (S)
Cefalexin (30 μg)	≤ 14	15–17	≥ 18
Ampicillin (10 μg)	≤ 13	14–16	≥ 17
Cephalothin (30 μg)	≤ 17	18–20	≥ 21
Ceftazidime (30 μg)	≤ 17	18–21	≥ 22
Cefotaxime (30 μg)	≤ 22	23–25	≥ 26
Cefepime (30 μg)	≤ 18	19–24	≥ 25
Amoxicillin (20 μg)	≤ 13	14–17	≥ 18
Meropenem (10 μg)	≤ 19	20–22	≥ 23
Penicillin (10 U)	≤ 17	18–20	≥ 21
Amikacin (30 μg)	≤ 14	15–17	≥ 18
Streptomycin (10 μg)	≤ 11	12–14	≥ 15
Kanamycin (30 μg)	≤ 13	14–17	≥ 18
Gentamicin (10 μg)	≤ 12	13–14	≥ 15
Tetracycline (30 μg)	≤ 14	15–18	≥ 19
Ciprofloxacin (5 μg)	≤ 20	21–30	≥ 31
Chloramphenicol (30 vμg)	≤ 12	13–17	≥ 18
Trimethoprim-sulfamethoxazole (20 μg)	≤ 10	11–15	≥ 16
Polymyxin B (300IU)	≤ 8	9–11	≥ 12

The evaluation criteria are primarily cited from CLSI M100 (32nd ed.), with reference to the standards therein that are applicable to human-derived bacteria.

### 2.5 MLST typing and analyses

MLST typing was conducted using Pasteur’s MLST scheme. We performed MLST typing utilizing a standardized protocol specific to *E. coli*, and eight housekeeping genes: dinB, icdA, pabB, polB, putB, trpA, trpB, and uidA. The primer information is listed in Supplementary File 1. Genomic DNA was extracted from *E. coli* isolates for PCR amplification under the following conditions: initial denaturation at 96°C for 1 min, followed by 30 cycles of denaturation (96°C for 1 min), annealing (55°C for 1 min), and extension (72°C for 1 min), with a final extension at 72°C for 10 min. The PCR products were sent to Nanjing Tsingke Biotechnology Co., Ltd. for sequencing. Upon completion of sequencing, the verified PCR product sequences were submitted to the *E. coli* MLST database^2^ to obtain the allele numbers of each housekeeping gene.

To analyze potential evolutionary relationships among the strains, the Phyloviz 2.0 software was employed to generate and visualize the minimum spanning tree (MST) and hierarchical clustering analysis results of *E. coli* strains (Feil et al., 2004). The geographical distribution and origins of *E. coli* ST479 were analyzed using global public MLST data of *E. coli* obtained from PubMLST database^3^

### 2.6 Whole-genome sequencing

In summary, bacterial genomic DNA was extracted using a bacterial genomic DNA extraction kit (DP302, TIANGEN, Beijing, China) following the manufacturer’s instructions. The quality of the DNA was assessed using a NanoDrop ND-1,000 spectrophotometer (ThermoFisher Scientific, Wilmington, United States). Qualified DNA samples were randomly sheared into fragments of approximately 350 bp using an ultrasonicator. The processed DNA fragments were subjected to the entire library preparation procedure using the NEBNext^®^ Ultra™ DNA Library Prep Kit for Illumina (NEB, United States), which included steps such as end repair, A-tailing, adapter ligation, purification, and PCR amplification. After library construction, the library was initially quantified using Qubit 2.0 and the insert size was verified using the Agilent 2100. Once the insert size met the expected criteria, the effective concentration of the library was accurately quantified using RT-qPCR to ensure library quality. Sequencing was subsequently performed using Illumina PE150 (NEB, United States).

The raw data were filtered and processed using the fastp software. After quality control of the original sequencing data, genome assembly was carried out by Unicycler software, and the assembly results were evaluated. Bacterial antibiotic-resistant genes and virulence genes were annotated through the Comprehensive Antibiotic Research Database 3.2.9^4^ and Virulence Factors of Pathogenic Bacteria Database,^5^ and statistics and analysis were carried out according to the information in the databases. Plasmid replicons were determined using Plasmid Finder 3.1 Database^6^ and plasmid replicons were typed (if applicable) using Plasmid MLST 2.0 Database^7^. Bacterial genome sequence was annotated with online tool Bakta 1.8.2 and visualized using Proksee 1.1.0.^8^

### 2.7 Cell culture and inflammatory response assay

BMECs were cultured in DMEM/F12 (Thermo Fisher, Waltham, MA, United States) supplemented with 10% fetal bovine serum and 1% ampicillin and streptomycin at 37°C in an atmosphere containing 5% carbon dioxide. Upon reaching approximately 80% confluence (approximately 10^^6^ cells/mL), ampicillin and streptomycin were removed from the culture, and the cells were incubated in six-well plates for 24 h. Subsequently, the cells were treated with *E. coli* (MOI 100:1), while the control group was treated with sterile PBS. Following an appropriate incubation period, cells were collected for subsequent analysis. Each treatment group consisted of three biological replicates.

### 2.8 Construction of mouse mastitis model

All experiments were conducted using lactating BALB/c female mice aged 9–12 weeks, with one mouse per cage. These mice had free access to food and water. Between postpartum days 5 and 7, mice with similar body weights were selected and randomly divided into three groups for treatment. In brief, overnight cultured bacteria were subcultured and propagated, with bacterial concentration determined through serial dilution and colony counting. A bacterial suspension was collected and centrifuged at 5,000 rpm for 5 min, followed by two washes and resuspensions in PBS to adjust the concentration to 5 × 10^^9^ CFU/mL. Subsequently, a 50 μL bacterial solution was directly injected into the fourth pair of mammary glands of the mice via the mammary duct. Control group mice received an equivalent injection of 50 μL sterile PBS solution. Model validation and criteria for success are as follows: The model was confirmed 24 h after injection by a combined assessment of mammary morphology, histopathology, and biochemical indicators. Only animals meeting all of the following predefined criteria were considered to have successful mastitis induction and were included in subsequent analyses: Clinical assessment: visual inspection of the injected fourth pair of mammary glands showing pronounced congestion (erythema) and swelling. Histopathological evaluation: excised mammary tissues fixed, sectioned, and stained with hematoxylin and eosin (H&E) to assess the degree of inflammatory cell infiltration in the mammary parenchyma. Increased relative expression of inflammatory genes in tissue: elevated expression of inflammatory genes such as TNF-α, IL-1β, and IL-6. Criteria for judgment based on prior research (Lee et al., 2003). There are three mice in each group, with a total of nine mice. The mice were monitored for 24 h, with body temperature measured hourly, followed by dissection to harvest mammary tissue. Each treatment group consisted of three biological replicates.

### 2.9 Total RNA extraction and quantitative RT-qPCR

Total RNA was extracted from cultured cells using Trizol reagent (Vazyme, Nanjing, China) according to the manufacturer’s instructions. Complementary DNA was synthesized using a reverse transcription kit (ThermoFisher Scientific, Wilmington, DE, United States). RT-qPCR experiments were conducted using SYBR Green PCR Master Mix (ThermoFisher Scientific, Wilmington, DE, United States) on the CFX Connect real-time PCR detection system (Bio-Rad, RI, United States). GAPDH was utilized as the house-keeping gene. The relative mRNA expression levels of the target genes were calculated using the 2^–Δ^ ^Δ^ *^Ct^* method. All primer sequences are listed in Supplementary File 2.

### 2.10 Cell viability assay

According to the manufacturer’s instructions, the Cell Counting Kit-8 (Beyotime, Shanghai, China) was used to detect cell viability. Briefly, BMECs were seeded in 96-well plates at a density of 2.5 × 10^3^ cells per well. Subsequently, *E. coli* was used for the attack. At the designated time points, the medium was replaced with 100 μL DMEM/F12 medium (supplemented with 10 μL CCK-8) and then incubated at 37 °C for 1.5 h. The absorbance at 450 nm was measured using a microplate reader (TECAN, Männedorf, Switzerland). All experiments were repeated three times.

### 2.11 Western blotting

Total protein from BMEC cells was harvested using the protein extraction kit (Beyotime, Shanghai, China). Proteins were separated using 10 or 15% SDS-PAGE and subsequently transferred to a 0.45 μm PVDF membrane. After blocking with 5% non-fat milk, the prepared PVDF membrane was incubated overnight at 4°C with specific primary antibodies, including NLRP6 (Cat No. 30973-1-AP), β-actin (Cat No. 20536-1-AP), TLR4 (Cat No. 19811-1-AP), NF-κB p65 (Cat No. 10745-1-AP), and Phospho-NF-κB p65 (Cat No. 80379-2-RR) sourced from Proteintech (Wuhan, China). Following three washes with TBST, the PVDF membrane was treated at room temperature for 2 h with goat anti-rabbit IgG (Beyotime, Shanghai, China). The membrane was washed three times with TBST. Finally, following the addition of enhanced chemiluminescence, the protein blots were imaged using the Western blotting detection system (Tanon, Shanghai, China).

### 2.12 Hematoxylin-eosin staining

Mammary tissue sections were dewaxed in xylene, and the slides were then hydrated in 95% ethanol for 5 min, 85% ethanol for 5 min, 75% ethanol for 5 min, and distilled water. Hematoxylin staining was then performed for 3 min, and then slides were rinsed with water for 2 min. After the runoff was transparent, the slides were sealed by the addition of a drop of neutral gum to the center of the paraffin section and adding a cover slip. The slides were then observed and photographed using a microscope (Leica, Germany).

### 2.13 Immunohistochemistry

The slides were incubated at 60°C for 30 min, dewaxed and hydrated. First, the sections were placed in xylene twice for 10 min each, incubated with 100, 95, 85, and 75% ethanol for 5–10 min and soaked in distilled water for 5 min. For antigen retrieval, the sections were incubated in citrate buffer (pH 6.0), heated in a microwave at high heat for 8 min, removed and cooled to room temperature. The slides were then incubated with the primary antibody, with the secondary antibodies in a box at 37°C for 1.5 h and then with a streptavidin-HRP antibody at 37°C for 20 min. After the runoff was transparent, the slides were incubated twice in xylene for 10 min each. Each slide was sealed with neutral gum and observed under the microscope.

### 2.14 Statistical analysis

All data were analyzed using GraphPad Prism 9.5.1 (San Diego, CA, United States) and expressed as mean ± standard deviation. Comparisons between two groups were conducted using the *t*-test, while comparisons involving more than two groups were performed using one-way analysis of variance to assess variance and test for significance. A significance level of *P* < 0.05 was considered statistically significant, with additional specific analyses detailed in the figure legends.

## 3 Results

### 3.1 Detection of antibiotic resistance of *E. coli*

**FIGURE 1:**
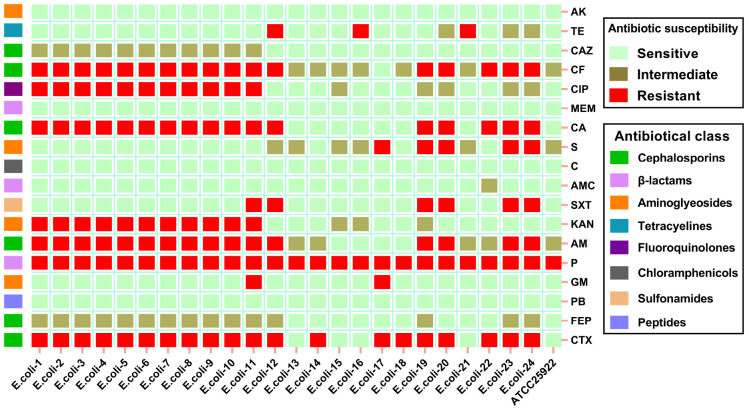
Antibiotic susceptibility testing of *E. coli*. Red indicates resistance to the drug, yellow represents intermediate susceptibility, and green signifies susceptible to the drug, *n* = 25. AK, Amikacin; AM, Ampicillin; AMC, Amoxicillin; C, Chloramphenicol; FEP, Cefepime; CA, Cefalexin; CAZ, Ceftazidime; CF, Cephalothin; CIP, Ciprofloxacin; CTX, Cefotaxime; GM, Gentamicin; KAN, Kanamycin; MEM, Meropenem; P, Penicillin; PB, Polymyxin B; S, Streptomycin; SXT, Trimethoprim-sulfamethoxazole; TE, Tetracycline.

The 16S rRNA sequencing results indicated the detection of 24 strains of *E. coli* among 96 milk samples. Through antibiotic susceptibility testing, 24 strains (100%), 20 strains (91.7%), and 17 strains (70.8%) of *E. coli* exhibited resistance to penicillin, cefotaxime, and cephalexin, respectively. Additionally, 23 strains (95.8%) and 20 strains (83.3%) displayed resistance or intermediate susceptibility to cephalothin and ampicillin, respectively. Furthermore, 15 strains (62.5%) and 11 strains (45.8%) of *E. coli* showed intermediate susceptibility to cefepime and ceftazidime, respectively. All isolated strains demonstrated no resistance to amikacin, chloramphenicol, meropenem, and polymyxin B (Figure 1). Notably, we observed a striking phenomenon where approximately 45% (*n* = 11) of *E. coli* exhibited remarkable consistency in their resistance profiles. Concurrently, these strains displayed resistance to a greater variety of antibiotics, resulting in 9 distinct resistance changes (7 antibiotic resistances, 2 intermediate susceptibilities) when compared to the laboratory strain ATCC25922. Subsequently, we conducted further typing analyses to determine whether these populations belonged to the same clonal group.

### 3.2 Molecular typing of *Escherichia coli* isolates

**FIGURE 2:**
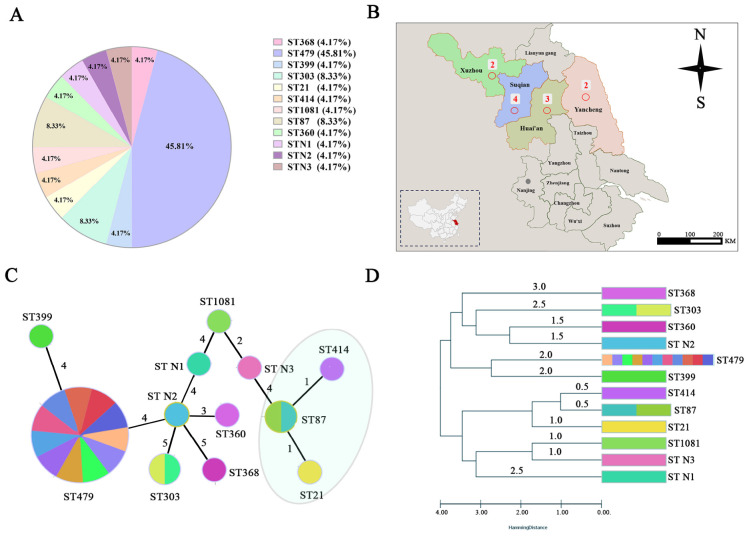
Molecular typing analysis of *E. coli*. **(A)** Results of MLST typing. **(B)** Geographical distribution of ST479. The numbers represent the quantity of ST479. The map lines delineate study areas and do not necessarily depict accepted national boundaries. **(C)** Minimum spanning tree of isolates (MST) . The MST was constructed based on CCs. The size of the circles is proportional to the number of isolates, and the host of the isolates was colored as indicated. The shadow zone in color represents a clone complexes, and the distances (connecting lines) between STs describe the number of allelic differences between them. **(D)** The phylogenetic tree constructed based on STs reveals four main groups. The number of colors represented at each node corresponds to the quantity of strains.

A total of 12 distinct sequence types (ST) were identified, with ST479 being the most prevalent (*n* = 11). The remaining STs included ST303 (*n* = 2), ST87 (*n* = 2), ST368 (*n* = 1), ST399 (*n* = 1), ST21 (*n* = 1), ST414 (*n* = 1), 1,081 (*n* = 1), 360 (*n* = 1), and three STs that were not assigned in the database (designated as N1, N2, and N3) (Figure 2A and Supplementary File 3). Consistent with our previous hypothesis, isolates exhibiting high levels of antibiotic resistance were all of the same ST, namely ST479. ST479 was derived from mastitis milk samples collected from four dairy farms in Jiangsu, China (Figure 2B), indicating that ST479 not only possesses extensive antibiotic resistance but also exhibits regional transmission advantages. Phylogenetic analysis revealed high genetic diversity among the *E. coli* isolates. Notably, the ST479 isolates formed a distinct, tightly clustered group, which was clearly separated from the other, more diverse isolates (Figures 2C,D). To investigate the origin and transmission routes of this clone, we conducted further analysis on ST479.

### 3.3 The global geographical dissemination of st479

**FIGURE 3:**
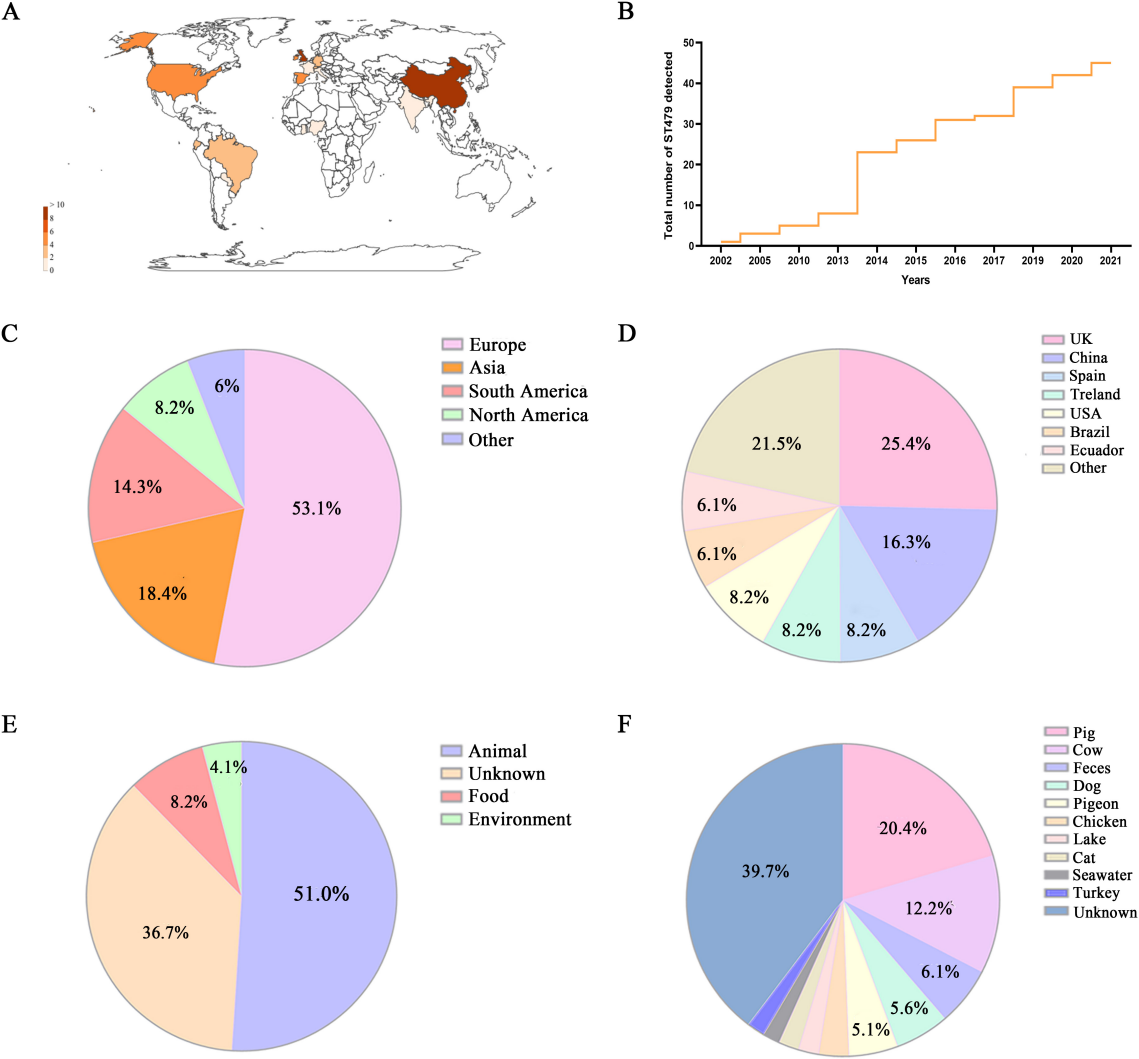
Global geographic distribution characteristics of ST479 from 2002 to 2022. **(A)** Heatmap of the number of ST479 strains detected in various regions worldwide. **(B)** Number of ST479 detections by year. **(C,D)** Countries and regions where ST479 has been detected. **(E,F)** Composition of ST479 strain sources and distribution of animal hosts.

The analysis of PubMLST public data indicates that ST479 is widely distributed globally, with Europe and Asia, particularly the United Kingdom and China, being the primary regions of distribution (Figure 3A). In the Pasteur MLST scheme, ST479 corresponds to ST224 in the Achtman MLST scheme. Our results show that ST479/224 was first detected and recorded by researchers in France in 2002. Since then, it has been continuously identified across various regions worldwide from 2002 to 2022, with a notable increase in detection rates occurring in 2014 (Figure 3B). Overall, *E. coli* ST479/224 has spread to varying extents in multiple countries, with the highest prevalence observed in the United Kingdom (25.4%), followed by China (16.3%), Spain (8.2%), Ireland (8.2%), and the United States (8.2%) (Figures 3C,D). Interestingly, 51% of ST479 was found in animal hosts, predominantly in pigs (20.4%) and cattle (12.2%) (Figures 3E,F).

### 3.4 Analysis of antibiotic resistance genes and virulence genes

**FIGURE 4:**
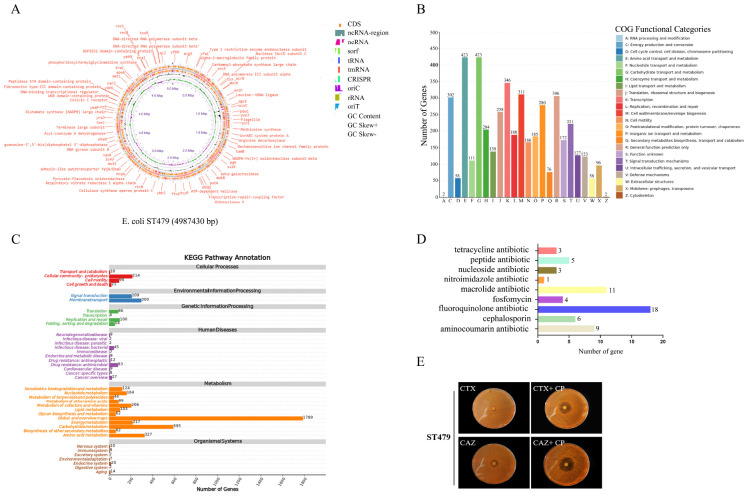
Genomic features of *E. coli* ST479. **(A)** Circular representation of the *E. coli* ST479 gene sequence. **(B,C)** COG function classification of 4574 core gene clusters. **(D)** Classification of antibiotic resistance genes contained in ST479. **(E)** Antibiotic sensitivity testing for ESBL production. CTX, Cefotaxime; CAZ, Ceftazidime; CP, Clavulanic acid.

The bacterial genome annotation reveals the 4987430-base pair sequence of *E. coli* ST479 (Figure 4A). The genomic annotation results indicate that ST479 contains 153 virulence genes, primarily focused on bacterial Adherence (*n* = 50), Effector delivery system (*n* = 29), and Motility (*n* = 48) according to the categories in The Virulence Factor Database (Supplementary File 4). More than 40 genes are associated with the bacterial pilus system, such as the stg ADCD gene cluster and the flgABCDEFGHIJKL gene cluster. These genes are primarily related to the rapid colonization and invasion of bacteria within the host. Additionally, several gene clusters associated with enterobactin synthesis and transport have been detected, including entABCDEF and fepABCD. Similar to most other MPEC isolates, ST479 possesses the type II secretion system (T2SS), type III secretion system (T3SS), and type VI secretion system (T6SS), as well as genes related to long polar fimbriae and iron capture (Table 2). The functional classification of 4,574 core genes in *E. coli* ST479 was explored using the COG database, revealing that most of these genes are involved in fundamental bacterial structure and metabolism (Figure 4B). The results of the KEGG enrichment analysis indicate that *E. coli* ST479 possesses a substantial number of genes associated with the Signal Transduction and Membrane Transport pathways, which are critically important for MPEC during infection of the bovine mammary gland (Figure 4C).

**TABLE 2:** Virulence genes of ST479.

Category	Subcategory	Number of VFs
Adherence		50
	Fimbrial adhesin	31
	Non-fimbrial adhesin	19
Effector delivery system		29
	Type II secretion system	9
	Type III secretion system	5
	Type VI secretion system	15
Invasion	Ibes	2
Motility	Flagella-mediated motility	48
Exotoxin	Colicin Ia	1
Immune modulation	LPS	4
Nutritional/Metabolic factor		14
	Metal uptake	4
	Metabolic adaptation	10
Regulation	RpoS	1
Others		4
Total		153

ST479 contains 60 resistance genes, predominantly comprising tetracycline antibiotics (*n* = 3), peptide antibiotics (*n* = 5), nucleoside antibiotics (*n* = 3), nitroimidazole antibiotics (*n* = 1), macrolide antibiotics (*n* = 11), fosfomycin (*n* = 4), fluoroquinolone antibiotics (*n* = 18), cephalosporins (*n* = 6), and aminocoumarin antibiotics (*n* = 9) (Figure 4D and Supplementary File 5). This includes the CTX-M-55 and aph(3’)-III genes, with the CTX-M-55 enzyme belonging to the CTX-M-1 group, first identified in *E. coli* producing CTX-M-55 type ESBLs in Thailand in 2004 (Kiratisin et al., 2007). Since then, it has rapidly spread to dozens of countries, including China, Japan, the USA, the UK, and Russia (Yang et al., 2023). Consequently, we conducted susceptibility tests to verify the production of CTX-M-type ESBLs. The results show that ST479 is a bla_CTX–M–55_-positive *E. coli* capable of producing ESBLs (Figure 4E).

In the genomic annotation of ST479, we identified two types of plasmids, pCoo and pECOE, belonging to the Inc.FII and Inc.B/O/K/Z types. The annotation results reveal that the plasmid pCoo contains the aph(3’)-III resistance gene and seven virulence genes: EspC, tssM, espP, pilV, Pic, pilN, and set1A. The pECOED plasmid did not annotate any resistance genes but contains two virulence genes, SenB and tssM.

### 3.5 Comparative pathogenic effects of ST479 and ATCC 25922 in experimental models

**FIGURE 5:**
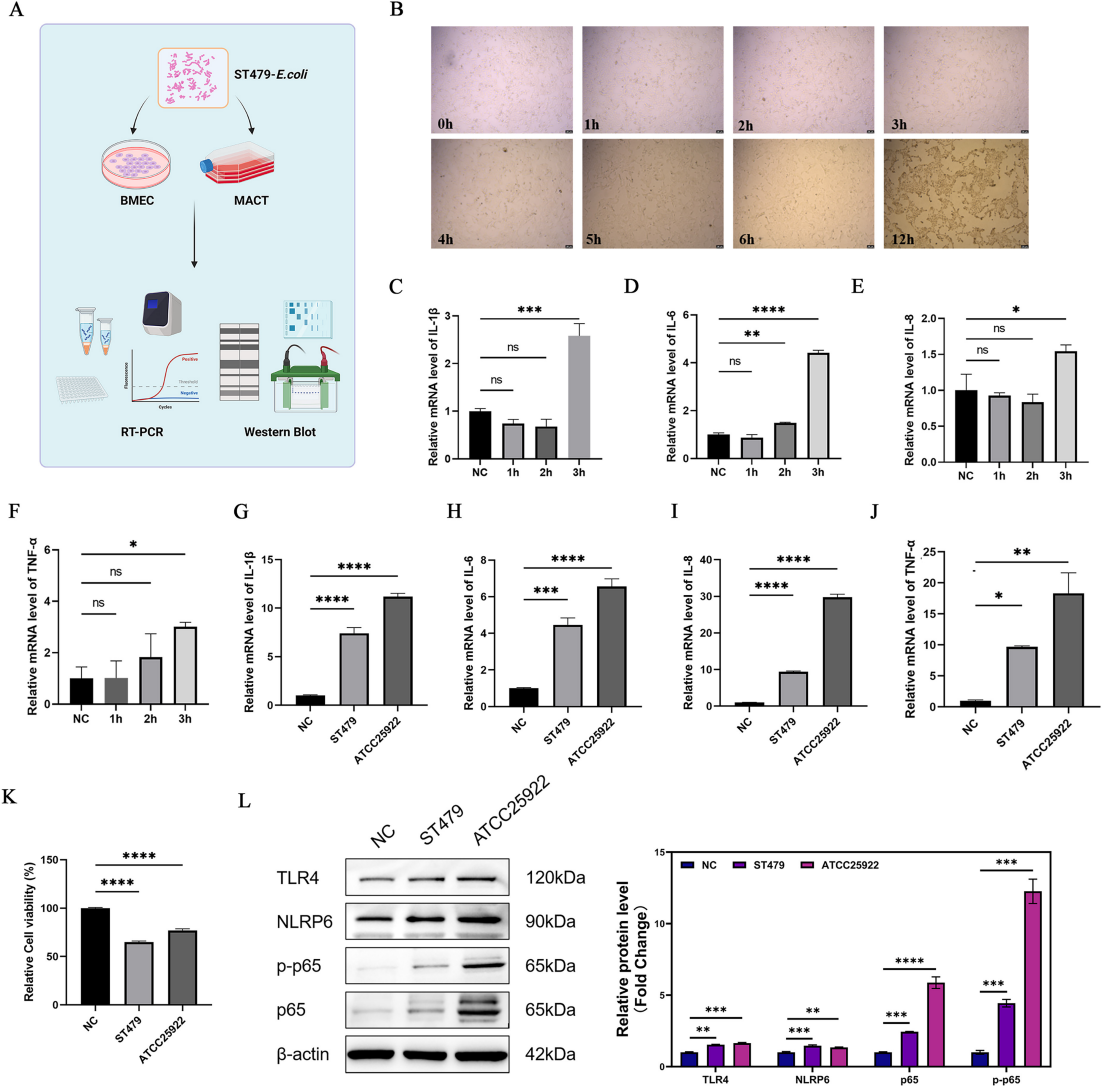
Pathogenic effects of *Escherichia coli* ST479 on BMECs. **(A)** The schematic diagram illustrating the induction mechanism of the inflammatory model in BMEC. **(B)** Microscopic images recorded over a 0–12-h time course. **(C–F)** mRNA levels of cellular inflammatory cytokines *IL-1β*, *IL-6*, *IL-8*, and *TNF-α* measured from 0 to 3 h. **(G–J)** mRNA levels of cellular inflammatory cytokines IL–1β, IL–6, IL–8, and TNF-α induced by ST479 and ATCC25922. **K**. Results of the cell viability assay. **L**. Western blotting analysis of NLRP6, TLR4, *p*-*p*65, *p*65 and β-actin expression, and grayscale value quantification. *^ns^*Not significant; *P < 0.05; ^**^P < 0.01; ***P < 0.001; ****P < 0.0001; *n* = 3.

To compare the pathogenic effects of ST479 with those of the reference strain ATCC 25922, we developed inflammation models using BMECs and the mouse mastitis model (Figure 5A). After challenge with ST479, the morphology of BMECs displayed significant changes at 3 h post-infection, and by 12 h, there was complete cell death (Figure 5B). RT-qPCR results indicated a marked increase in the mRNA levels of inflammatory cytokines IL-1β, IL-6, IL-8, and TNF-α in BMECs at 3 h post-infection (Figures 5C–F). In comparison to the laboratory strain ATCC29522, the upregulation of inflammatory cytokine mRNA was lower in cells infected with ST479 (Figures 5G–J). However, there was a more significant decline in cell viability (Figure 5K). This suggests that alterations in the virulence genes and antibiotic resistance of ST479 may have modified its pathogenic capabilities and mechanisms relative to ATCC25922. The reduced inflammatory response likely enhances the host’s chances of survival, thereby facilitating the growth and dissemination of ST479. Furthermore, Western blotting results demonstrated that, similar to ATCC25922, ST479 activates the classical inflammatory signaling pathways NLRP6/TLR4/NF κB following the induction of inflammation (Figure 5L).

**FIGURE 6:**
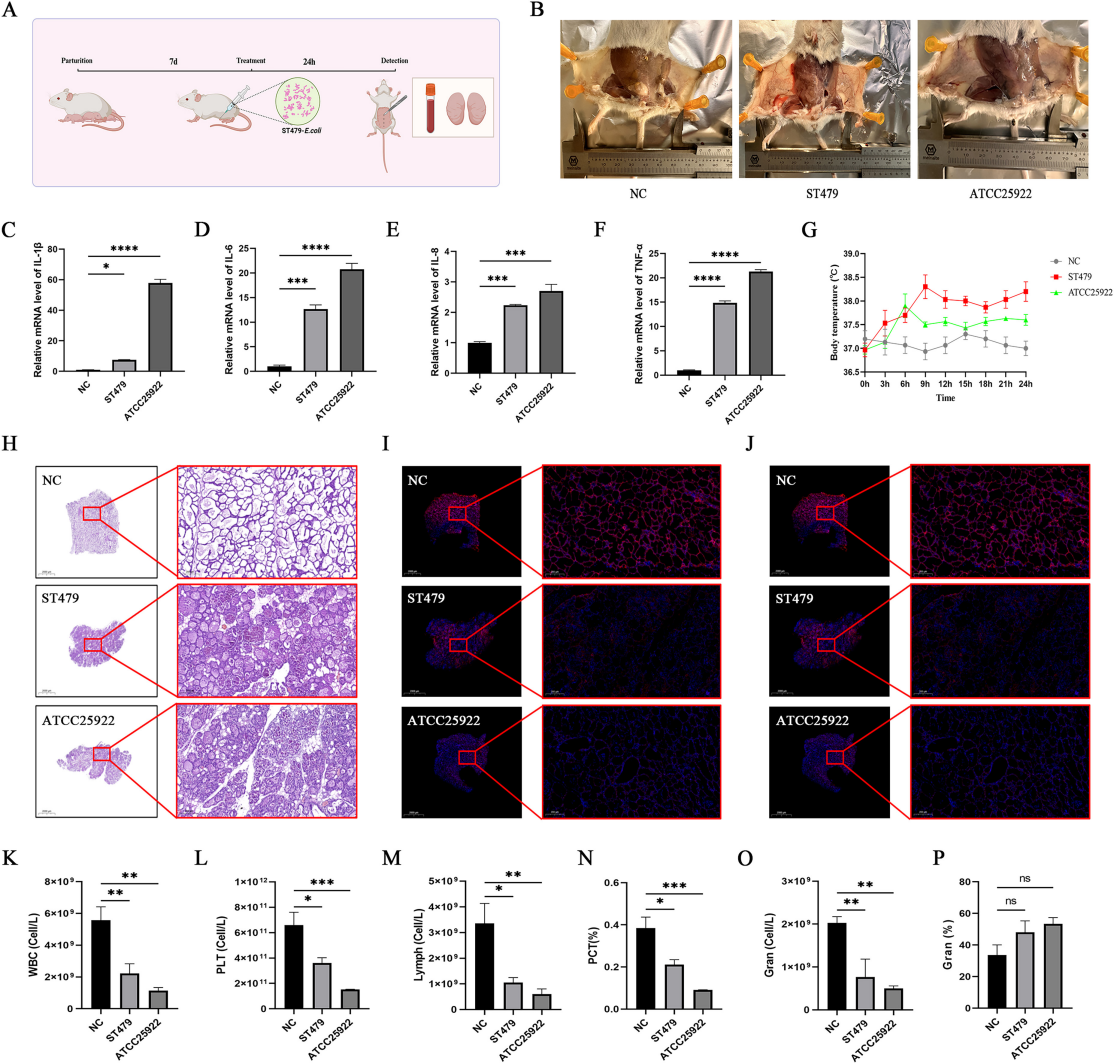
The pathogenic role of *Escherichia coli* ST479 in mouse mastitis. **(A)** Schematic diagram of the induction mechanism of the mouse mastitis model. **(B)** Photographs of mouse mammary tissue. **(C–F)** mRNA expression levels of inflammatory cytokines *IL-1β*, *IL-6*, *IL-8*, and *TNF-α* in mouse mammary tissue. **(G)** Body temperature measurements of mice over 0–24 h. **(H)** Hematoxylin and eosin (H&E) staining images of mouse mammary tissue. **(I,J)** Immunofluorescence images of tight junction proteins ZO-1 and Occludin in mouse mammary tissue. **(K–P).** Analysis of routine blood parameters. *^ns^*Not significant; *P < 0.05; **P < 0.01; ***P < 0.001; ****P < 0.0001; *n* = 3.

A murine mastitis model was established to comparatively assess the pathogenic potential and host inflammatory response induced by *E. coli* ST479 against the reference strain ATCC 25922. The mouse model of mastitis was successfully established, similar to the model of BMECs. Results indicated that 24 h after induction with ST479, the mammary tissues of the mice showed diffuse hemorrhage, tissue pallor, and other inflammatory symptoms (Figure 6B). Additionally, the body temperature increased rapidly and was significantly higher than that of the control group (Figure 6G). RT-qPCR analysis of mammary tissues revealed that the mRNA levels of inflammatory cytokines IL–1β, IL–6, IL–8, and TNF-α were significantly elevated (*P* < 0.05) (Figures 6C–F). H&E staining demonstrated that the morphology of the mouse mammary tissue was compromised, showing notable damage to the alveolar lumen structure and extensive infiltration of inflammatory cells (Figure 6H). Immunofluorescence imaging revealed a reduction in tight junction proteins ZO-1 and Occludin in the mammary glands of treated mice compared to the control group (Figures 6I,J). Complete blood count results indicated that mice with mastitis exhibited decreased numbers of white blood cells, platelets, lymphocytes, and platelet volume fraction (*P* < 0.05) (Figures 6K–N). Interestingly, although the percentage of neutrophils showed an upward trend, the absolute number of neutrophils significantly decreased (*P* < 0.05) (Figures 6O,P). Furthermore, based on clinical symptoms, inflammatory cytokine mRNA levels, and routine blood test parameters, mastitis induced by ST479 in mice appeared to be less severe than that induced by the laboratory strain ATCC25922.

## 4 Discussion

Mastitis caused by *E. coli* in dairy cows presents a significant challenge to the dairy farming industry. The use of antimicrobials in animal husbandry can exert selective pressure for resistance. Although antimicrobial treatment is not generally recommended for mild to moderate cases of *E. coli* mastitis, which are often self-limiting, severe or toxic cases may require therapy (Suojala et al., 2013). It is important to note that many countries have established stringent regulations to govern antimicrobial use in dairy farming. Nonetheless, surveillance of resistance patterns remains crucial to evaluate the effectiveness of such stewardship measures and to address challenges in regions or production systems where such practices may persist.” Our study focused on isolating and identifying *E. coli* from milk samples collected from dairy cows with clinical mastitis in Jiangsu Province, China. Through multi-locus sequence typing, antimicrobial susceptibility testing, and whole-genome sequencing, we examined the resistance, evolution, and distribution characteristics of *E. coli* in farms throughout Jiangsu. We identified a multidrug-resistant, ESBL-producing MPEC clone (ST479).

Our results indicate that *E. coli* isolates from bovine mastitis milk samples in Jiangsu, China, exhibit resistance to most β-lactam antibiotics, including first-, third-, and fourth-generation cephalosporins, while remaining susceptible to the majority of aminoglycoside antibiotics. The observation that approximately 45% of isolates shared an identical, broad resistance profile led us to hypothesize that the emergence of multidrug resistance, possibly coupled with clonal expansion, contributed to this consistency. Subsequent molecular typing confirmed that these isolates indeed belonged to the same sequence type (ST479), supporting this interpretation. ST479 exhibit resistance to antimicrobials spanning three or more classes (e.g., β-lactams, tetracyclines, sulfonamides, aminoglycosides, fluoroquinolones), meeting the standard criteria for classification as multidrug-resistant *E. coli* (Magiorakos et al., 2012). The isolation of a multidrug-resistant, ESBL-producing clone (ST479) as the predominant strain in our study highlights a potential challenge for mastitis management. While antimicrobial therapy is not routinely indicated for all cases of *E. coli* mastitis, the presence of resistance to multiple drug classes, including critically important cephalosporins, could compromise treatment efficacy in severe cases where intervention is necessary (Hickson et al., 2025). Furthermore, the clonal spread of such resistant strains within and between farms, as suggested by our MLST data, underscores the importance of monitoring resistance patterns to inform stewardship and biosecurity measures.

Global dissemination analysis indicates that ST479 isolates emerged worldwide approximately two decades ago, with the earliest records found in France. This trend aligns with the widespread introduction and use of broad-spectrum cephalosporins, which have exerted substantial selective pressure. Global source distribution analysis indicates that ST479 is not only widely distributed across different regions of the world but also predominantly associated with animal populations. Over the past twenty years, ST479/224 strains have spread globally, with reports from multiple countries and regions, particularly in Europe and Asia, where they have been identified among various animal hosts (Liu et al., 2013). Researchers in Poland have documented the presence of ST479 in both hospital and community settings, with isolates carrying the bla_KPC–3_ gene (Ojdana et al., 2015). In Brazil, a study identified CTX-M-15-producing *E. coli* ST479/224 strains in commercial pigs (Silva et al., 2016).

Many studies have described virulence genes associated with MPEC, but until now there has been no specific virulence genes have been identified that can accurately characterize MPEC strains (Guerra et al., 2020; Orsi et al., 2023). Gene annotations have revealed that T2SS, T3SS, and T6SS are the primary bacterial secretion systems in *E. coli* ST479, along with various virulence genes related to invasion, adhesion, iron acquisition, and immunity. Some studies have explored the distribution of virulence genes in MPEC across different phylogenetic groups (Li et al., 2023). However, due to factors like sample size, MPEC isolates show considerable genetic heterogeneity, and no specific genetic pattern for MPEC has been identified (Wyrsch et al., 2022). There is a need for further research on the virulence factors of MPEC to determine whether it has unique characteristics that set it apart from other types of *E. coli*. Research has indicated that certain *E. coli* genes, such as traT, ecpA, and ompT, could serve as biomarkers for the clinical severity of bovine mastitis (Guerra et al., 2020). While this study investigated the diversity of virulence factors, it did not examine the expression levels of these factors, which represents a limitation of the research.

Our results indicate that ST479 is a bla_CTX–M–55_-positive *E. coli* strain capable of producing ESBLs, which explains its resistance to most β-lactam antibiotics. Studies have shown that among *E. coli* isolates resistant to third-generation cephalosporins, the proportion that produce broad-spectrum β-lactamases ranges from 65 to 100%. The CTX-M type is the most prevalent among these ESBLs (Arlet et al., 2006). The global use of antibiotics has undoubtedly introduced new dynamics that are altering bacterial populations. These changes may not only modify the characteristics of bacterial pathogens but also potentially transform the properties of the normal host microbiota (Martínez and Baquero, 2002). In recent years, bla_CTX–M–55_-positive *E. coli* has been frequently reported in both clinical patients and farm animals, showing a rapid increase and posing significant risks to human health and agricultural productivity (Norizuki et al., 2018). In 2017, the World Health Organization identified a priority list of antimicrobial-resistant organisms for research, categorizing ESBL-producing Enterobacteriaceae within this priority group. CTX-M-55 was first identified in Thailand in 2004 and has since become the most common CTX-M subtype found in animal-derived *E. coli* in China (Zhang et al., 2014). The global prevalence of CTX-M-55-producing ESBL Enterobacteriaceae is on the rise, and the mechanisms of transmission remain not fully understood, thereby threatening the health of both the public and livestock.

The ST479 clone poses a dual threat to the dairy industry, manifesting at both the individual-animal treatment level and the herd-level transmission dynamics. At the individual-treatment level, its multidrug-resistant phenotype represents a clear clinical challenge. ST479 exhibits resistance to multiple commonly used agents, including third-generation cephalosporins, and harbors CTX-M-55–type extended-spectrum β-lactamases, which substantially constrain first-line therapeutic options. Therapeutic failure or delays in administering effective treatment may prolong disease duration, increase milk-yield losses, culling risk, and veterinary intervention costs, and thereby directly impair animal welfare and farm-level economic outcomes. At the herd level, the clonal nature of ST479 indicates a potential for persistent dissemination. In this study ST479 emerged as a dominant clone across geographically distinct farms, and global PubMLST data reveal its detection in multiple countries and in diverse animal hosts (notably cattle and swine), strongly suggesting enhanced adaptability and transmissibility. Resistance determinants (e.g., bla_CTX–M–55_) and virulence genes located on plasmids within its genome may spread and become fixed in bacterial populations via horizontal gene transfer or clonal expansion. Consequently, once such a resistant clone becomes established in a farm environment it may be difficult to eradicate and can serve as a regional reservoir of resistance genes, posing a “genetic contamination” risk to other bacteria and even other pathogens. Therefore, concern about multidrug-resistant clones such as ST479 extends beyond their infection-causing capacity to include their potential to compromise treatment options and sustain transmission within herds. This underscores the importance of implementing molecular typing–based targeted surveillance in dairy production. Monitoring the dynamics of such clones enables earlier detection of spread, assessment of infection-control interventions, and provides critical evidence to inform risk-based, precision biosecurity and antimicrobial stewardship strategies at the farm level.

The comparative analysis between the predominant *E. coli* ST479 clone and the reference strain ATCC 25922, conducted in both bovine mammary epithelial cells (BMECs) and a standardized murine mastitis model, revealed a distinct pathogenic profile for ST479. ST479 has the capacity to elicit BMEC responses and induce inflammation in murine mammary tissue, and, like ATCC25922, activates the canonical NLRP6/TLR4/NF-κB signaling pathway. However, compared with ATCC25922, ST479 provokes a comparatively attenuated host inflammatory response despite harboring a broader repertoire of antibiotic resistance and virulence genes. Notably, the attenuated inflammatory profile observed in the mouse mastitis model-characterized by moderated cytokine responses and altered hematological parameters-was consistent with the reduced pro-inflammatory signature observed in ST479-infected BMECs. This attenuated inflammatory phenotype, observed alongside its multidrug resistance, may reflect an evolutionary adaptation that balances effective colonization with host immune modulation, potentially enhancing bacterial survival and dissemination within host populations. The inverse relationship observed between heightened antimicrobial resistance and moderated virulence in ST479 aligns with the concept that selective pressures can shape bacterial fitness (Guillard et al., 2016). One interpretation is that within the bovine host, selection may favor strains that maintain infection without triggering overwhelming inflammation, thereby promoting bacterial shedding and transmission while accommodating the metabolic burden of resistance determinants. This profile is consistent with the characterization of many mastitis-associated *E. coli* isolates as versatile commensals with pathogenic potential (Suojala et al., 2011), where genetic adjustments may facilitate evasion of host immune surveillance rather than outright confrontation. An alternative explanation is that specific genetic adaptations in MPEC may enhance their ability to evade or modulate the bovine host’s immune response. The severity of *E. coli* mastitis is a multifactorial outcome, shaped by the interplay of pathogen virulence and host susceptibility. The incidence and severity of clinical *E. coli* mastitis peak during the postpartum and early lactation periods, a phase characterized by physiological stress and relative immunosuppression. Other cow-level factors such as parity, metabolic status, and genetic predisposition also significantly influence disease manifestation (Burvenich et al., 2003). Therefore, the clinical outcome likely results from the interaction between a susceptible host and a pathogen possessing a specific, and potentially adapted, virulence repertoire. The ecological significance of this moderated inflammatory phenotype in the natural bovine host remains speculative and warrants further investigation. It may reflect adaptations unrelated to disease severity but perhaps to persistence or transmission. While the ecological and clinical implications of this phenotype in cattle require further investigation, it highlights the diversity of host-pathogen interactions among mastitis-associated *E. coli.*

This study has several limitations that should be considered. First, methodological adaptations were necessary to serve dual objectives. While our bacteriological protocol strictly followed NMC guidelines for contamination assessment (excluding samples with > 2 morphologies), the parallel use of serial dilution for isolating single clones represents a deviation from pure diagnostic procedures. This step was essential for obtaining genetically homogeneous material for WGS and MLST but may not reflect the standard isolation workflow in all veterinary diagnostic laboratories. Second, the use of a murine mastitis model, while valuable for controlled comparative studies, has inherent limitations in directly extrapolating to the complex pathophysiology of bovine mastitis, which involves species-specific host factors, lactation physiology, and management environment. Third, the antimicrobial susceptibility testing employed breakpoints primarily derived from human medicine (CLSI M100) for agents lacking veterinary-specific standards. Although this is an accepted practice for surveillance, it may not perfectly correlate with clinical outcomes in cattle. Finally, our sampling focused on clinical mastitis cases of a defined severity (score ≥ 2), which may not capture the full diversity of *E. coli* populations involved in subclinical or milder infections.

The resistance profile of ST479 indicates that empiric use of β-lactam antibiotics, particularly third-generation cephalosporins, may be ineffective in populations where this clone is prevalent. This underscores the necessity of culture-guided therapy for severe mastitis cases and highlights the importance of preserving the efficacy of last-resort agents (such as carbapenems, to which ST479 remains susceptible). The widespread geographic distribution of ST479 and increasing detection over time in multiple regions necessitate a reassessment of biosafety and control strategies. Enhanced preventative measures addressing transmission via milking equipment, personnel, and environmental contamination should be considered. Strict isolation and management of cattle infected with multidrug-resistant pathogens may be a key strategy to limit dissemination. Therefore, future studies should employ epidemiological approaches to track pathogenic, widely distributed, multidrug-resistant lineages such as ST479. This should include longitudinal on-farm investigations to understand their persistence, and the application of genomic epidemiology to trace transmission between farms, among different livestock species (for example, cattle-to-swine spread suggested by concurrent presence in both cattle and pigs), and potentially across the human–animal interface. Studying the plasmid ecology of elements carrying bla_CTX–M–55_ and other resistance genes is essential for assessing the risk of horizontal transfer of these genes to other pathogens. From a public-health perspective, the detection of ESBL-producing clones in dairy cattle and their global distribution warrants attention within a One Health framework. Future research should evaluate the potential for zoonotic transmission via direct contact, environmental shedding, or the food chain. Ultimately, integrating genomic surveillance data from animal, human, and environmental sources will be key to understanding the full lifecycle of such resistant clones and to designing effective interventions.

Antibiotic resistance in bacteria is a global concern, and globalization, along with the movement of people, animals, and food, contributes to the worldwide spread of resistant bacteria. To effectively track the dissemination of antibiotic resistance, it is essential to implement monitoring programs and analyze various food categories to detect changes in the distribution of resistant bacteria, resistance genes, and plasmids. Additionally, further research on the virulence mechanisms, phylogenetic groups, and antimicrobial resistance patterns of multidrug-resistant MPEC is necessary to better understand the epidemiology and pathogenicity of *E. coli* in bovine mastitis. To our knowledge, this is the first report in China of bla_CTX–M–55_ in *E. coli* isolated from milk samples of mastitis in Holstein cows. The emergence of multidrug-resistant MPEC in cases of bovine mastitis highlights the urgent need for enhanced and continuous monitoring of antimicrobial resistance in bacterial isolates from the livestock industry. It also emphasizes the importance of issuing alerts to prevent the rise of highly invasive pandemic strains and to avoid failures in antimicrobial treatments.

## Data Availability

The datasets generated and analyzed during this study can be found in the NCBI BioProject database repository under accession number PRJNA1291126. The datasets used and analyzed during the current study are available from the corresponding author on reasonable request.

## Footnotes

1
https://blast.ncbi.nlm.nih.gov/Blast.cgi

2
https://bigsdb.pasteur.fr/ecoli/

3
https://pubmlst.org/

4
https://card.mcmaster.ca/

5
www.mgc.ac.cn/VFs/

6
https://cge.food.dtu.dk/services/PlasmidFinder/

7
https://cge.food.dtu.dk/services/pMLST/

8
https://proksee.ca/
