# Supplementary material for: Multidrug-resistant mammary pathogenic Escherichia coli ST479 isolated from Holstein dairy cows in Jiangsu, China
Source: Front Microbiol. 2026 Mar 3;17:1737656. doi: 10.3389/fmicb.2026.1737656 (PMC13067290; doi:10.3389/fmicb.2026.1737656)
Supplement: Supplementary file 1 [file Table_1.DOCX]

**Additional file 1. The Primers for MLST Typing and Identification of *Escherichia coli.***

| Gene | Primer name | Primer sequence |
| --- | --- | --- |
| 16s | Forward | AGAGTTTGATCCTGGCTCAG |
|  | Reverse | TACGGCTACCTTGTTACGACTT |
| dinB | dinB-F  dinB-R | GTTTTCCCAGTCACGACGTTGTATGAGAGGTGAGCAATGCGTA  TTGTGAGCGGATAACAATTTCCGTAGCCCCATCGCTTCCAG |
| icdA | icdA-F  icdA-R | GTTTTCCCAGTCACGACGTTGTAATTCGCTTCCCGGAACATTG  TTGTGAGCGGATAACAATTTCATGATCGCGTCACCAAAYTC |
| pabB | pabB-F  pabB-R | GTTTTCCCAGTCACGACGTTGTAAATCCAATATGACCCGCGAG  TTGTGAGCGGATAACAATTTCGGTTCCAGTTCGTCGATAAT |
| polB | polB-F  polB-R | GTTTTCCCAGTCACGACGTTGTAGGCGGCTATGTGATGGATTC  TTGTGAGCGGATAACAATTTCGGTTGGCATCAGAAAACGGC |
| putB | putB-F  putB-R | GTTTTCCCAGTCACGACGTTGTACTGTTTAACCCGTGGATTGC  TTGTGAGCGGATAACAATTTCGCATCGGCCTCGGCAAAGCG |
| trpA | trpA-F  trpA-R | GTTTTCCCAGTCACGACGTTGTAGCTACGAATCTCTGTTTGCC  TTGTGAGCGGATAACAATTTCGCTTTCATCGGTTGTACAAA |
| trpB | trpB-F  trpB-R | GTTTTCCCAGTCACGACGTTGTACACTATATGCTGGGCACCGC  TTGTGAGCGGATAACAATTTCCCTCGTGCTTTCAAAATATC |
| uidA | uidA-F  uidA-R | GTTTTCCCAGTCACGACGTTGTACATTACGGCAAAGTGTGGGTCAAT  TTGTGAGCGGATAACAATTTCCCATCAGCACGTTATCGAATCCTT |
