# Supplementary material for: Multidrug-resistant mammary pathogenic Escherichia coli ST479 isolated from Holstein dairy cows in Jiangsu, China
Source: Front Microbiol. 2026 Mar 3;17:1737656. doi: 10.3389/fmicb.2026.1737656 (PMC13067290; doi:10.3389/fmicb.2026.1737656)
Supplement: Supplementary file 2 [file Table_2.DOCX]

**Additional file 2. The Sequences of primers used for RT-qPCR.**

| **Specie** | **Gene** | **Primer name** | **Primer sequence** |
| --- | --- | --- | --- |
| mouse | *IL1β* | IL1β-F  IL1β-R | ATGCCACCTTTTGACAGTGATG  TGTGCTGCTGCGAGATTTGA |
|  | *IL6* | IL6-F  IL6-R | AGCCAGAGTCCTTCAGAGAGA  GCCACTCCTTCTGTGACTCC |
|  | *IL8* | IL8-F  IL8-R | CTAGGCATCTTCGTCCGTCC  TTCACCCATGGAGCATCAGG |
|  | *TNF-α* | TNF-α-F  TNF-α-R | GATCGGTCCCCAAAGGGATG  CCACTTGGTGGTTTGTGAGTG |
|  | *GAPDH* | GAPDH-F  GAPDH-R | TGTGTCCGTCGTGGATCTGA  TTGCTGTTGAAGTCGCAGGAG |
| bos | *IL1β* | IL1β-F  IL1β-R | ATCTATACCTGTCTTGTG  TTCTTGATTTCTGTCTTG |
|  | *IL6* | IL6-F  IL6-R | AGAACGAGTATGAGGGAAAT  TGGCTGGAGTGGTTATTAG |
|  | *IL8* | IL8-F  IL8-R | AAGAATTGAGAGTTATTGAGAGT  CAGACCTCGTTTCCATTG |
|  | *TNF-α* | TNF-α-F  TNF-α-R | ATTAGGGATGTAGGGAAGTGAGG  CAAGGAATGTTGCGAAGTGTT |
|  | *GAPDH* | GAPDH-F  GAPDH-R | GGCAAGTTCAACGGCACA  ACCACATACTCAGCACCAGCA |
