# Supplementary material for: Multidrug-resistant mammary pathogenic Escherichia coli ST479 isolated from Holstein dairy cows in Jiangsu, China
Source: Front Microbiol. 2026 Mar 3;17:1737656. doi: 10.3389/fmicb.2026.1737656 (PMC13067290; doi:10.3389/fmicb.2026.1737656)
Supplement: Supplementary file 3 [file Table_3.DOCX]

**Additional file 3. MLST Allelic profile of *Escherichia coli*.**

| **Sample** | | **Allelic profile-Pasteur scheme** | | | | | | | | | | | | | | | | |
| --- | --- | --- | --- | --- | --- | --- | --- | --- | --- | --- | --- | --- | --- | --- | --- | --- | --- | --- |
|  |  | dinB | | icdA | | pabB | | polB | | putP | | trpA | | trpB | | uidA | | ST |
| *E.coli*-1 | 5 | | 37 | | 4 | | 10 | | 78 | | 8 | | 2 | | 30 | | 479 | |
| *E.coli*-2 | 5 | | 37 | | 4 | | 10 | | 78 | | 8 | | 2 | | 30 | | 479 | |
| *E.coli*-3 | 5 | | 37 | | 4 | | 10 | | 78 | | 8 | | 2 | | 30 | | 479 | |
| *E.coli*-4 | 5 | | 37 | | 4 | | 10 | | 78 | | 8 | | 2 | | 30 | | 479 | |
| *E.coli*-5 | 5 | | 37 | | 4 | | 10 | | 78 | | 8 | | 2 | | 30 | | 479 | |
| *E.coli*-6 | 5 | | 37 | | 4 | | 10 | | 78 | | 8 | | 2 | | 30 | | 479 | |
| *E.coli*-7 | 5 | | 37 | | 4 | | 10 | | 78 | | 8 | | 2 | | 30 | | 479 | |
| *E.coli*-8 | 5 | | 37 | | 4 | | 10 | | 78 | | 8 | | 2 | | 30 | | 479 | |
| *E.coli*-9 | 5 | | 37 | | 4 | | 10 | | 78 | | 8 | | 2 | | 30 | | 479 | |
| *E.coli*-10 | 5 | | 37 | | 4 | | 10 | | 78 | | 8 | | 2 | | 30 | | 479 | |
| *E.coli*-11 | 5 | | 37 | | 4 | | 10 | | 78 | | 8 | | 2 | | 30 | | 479 | |
| *E.coli*-12 | 50 | | 47 | | 3 | | 10 | | 5 | | 7 | | 98 | | 103 | | 368 | |
| *E.coli*-13 | 32 | | 47 | | 3 | | 10 | | 78 | | 8 | | 2 | | 2 | | 399 | |
| *E.coli*-14 | 7 | | 33 | | 18 | | 2 | | 5 | | 28 | | 2 | | 2 | | 21 | |
| *E.coli*-15 | 7 | | 164 | | 18 | | 2 | | 5 | | 8 | | 2 | | 2 | | 414 | |
| *E.coli*-16 | 7 | | 3 | | 4 | | 238 | | 5 | | 108 | | 58 | | 65 | | 1081 | |
| *E.coli*-17 | 82 | | 3 | | 4 | | 10 | | 71 | | 1 | | 98 | | 5 | | 360 | |
| *E.coli*-18 | 7 | | 3 | | 4 | | 25 | | 76 | | 108 | | 98 | | 30 | | N1 | |
| *E.coli*-19 | 25 | | 3 | | 48 | | 10 | | 26 | | 57 | | 98 | | 90 | | 303 | |
| *E.coli*-20 | 25 | | 3 | | 48 | | 10 | | 26 | | 57 | | 98 | | 90 | | 303 | |
| *E.coli*-21 | 5 | | 3 | | 4 | | 10 | | 5 | | 1 | | 98 | | 30 | | N2 | |
| *E.coli*-22 | 7 | | 3 | | 18 | | 238 | | 5 | | 108 | | 2 | | 65 | | N3 | |
| *E.coli*-23 | 7 | | 33 | | 18 | | 2 | | 5 | | 8 | | 2 | | 2 | | 87 | |
| *E.coli*-24 | 7 | | 33 | | 18 | | 2 | | 5 | | 8 | | 2 | | 2 | | 87 | |
