# Supplementary material for: Multidrug-resistant mammary pathogenic Escherichia coli ST479 isolated from Holstein dairy cows in Jiangsu, China
Source: Front Microbiol. 2026 Mar 3;17:1737656. doi: 10.3389/fmicb.2026.1737656 (PMC13067290; doi:10.3389/fmicb.2026.1737656)

1. The full uncropped images of the Western blot experimental results are as follows.

(1) β-actin


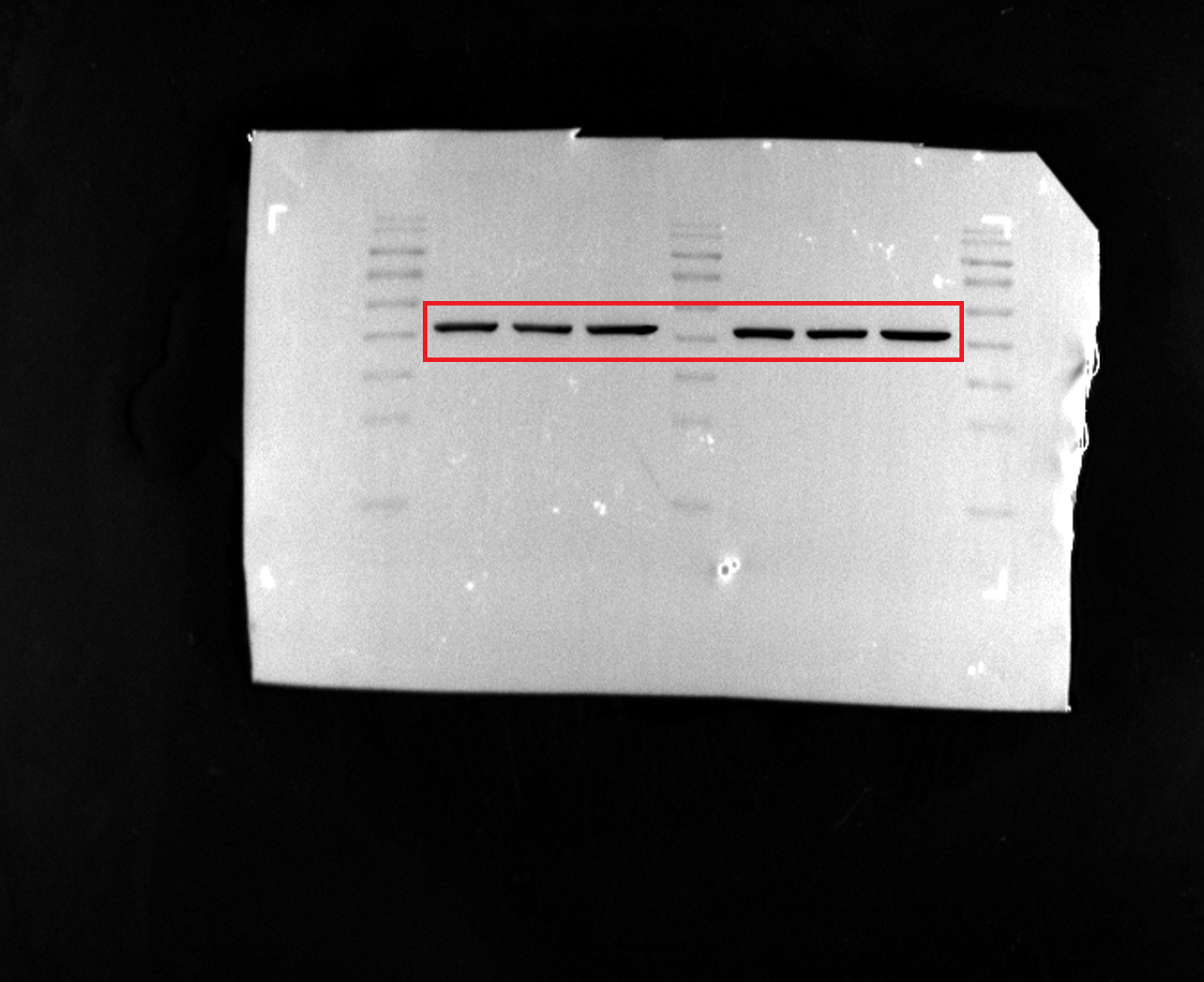


(2) p65


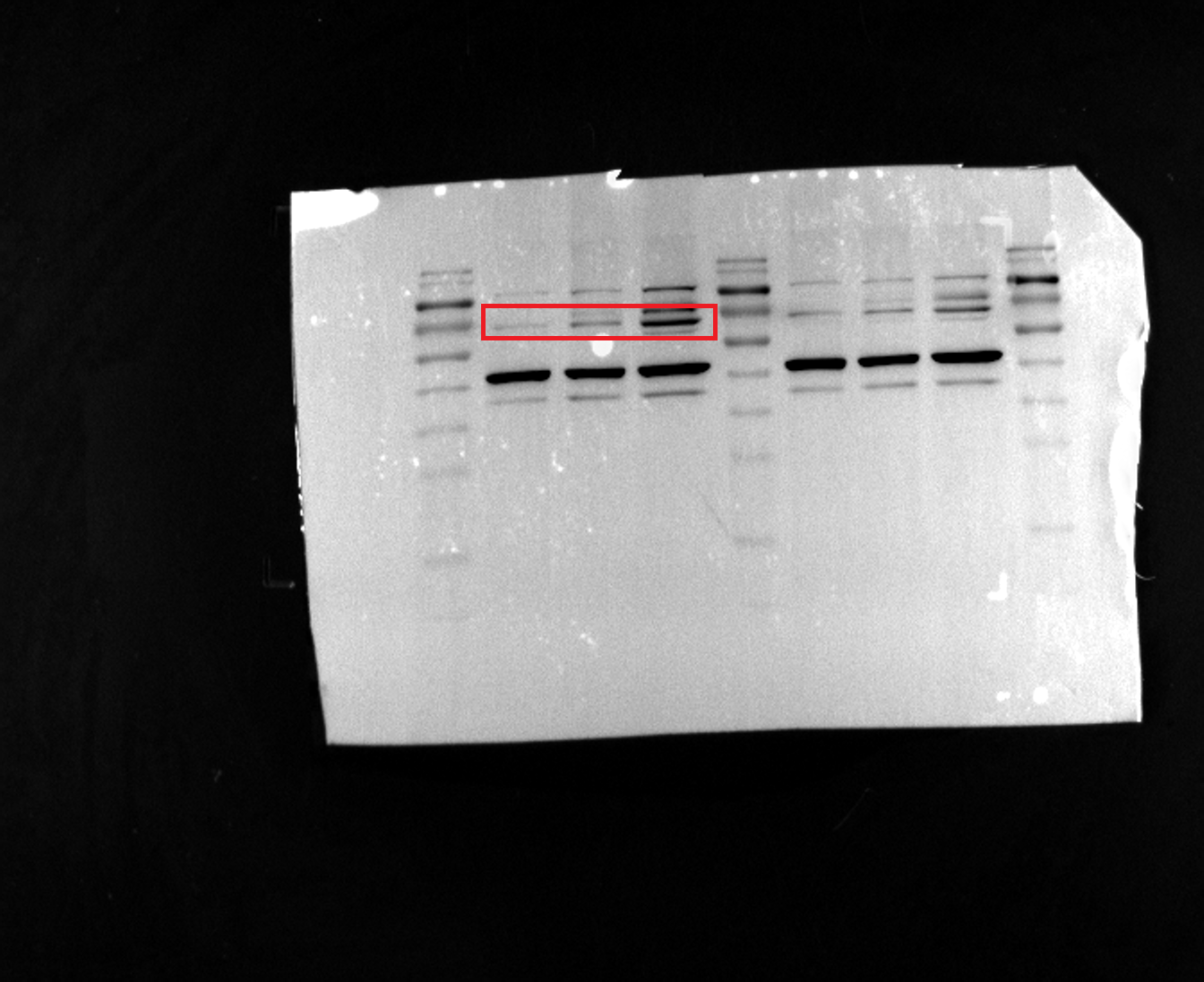


(3) p-p65


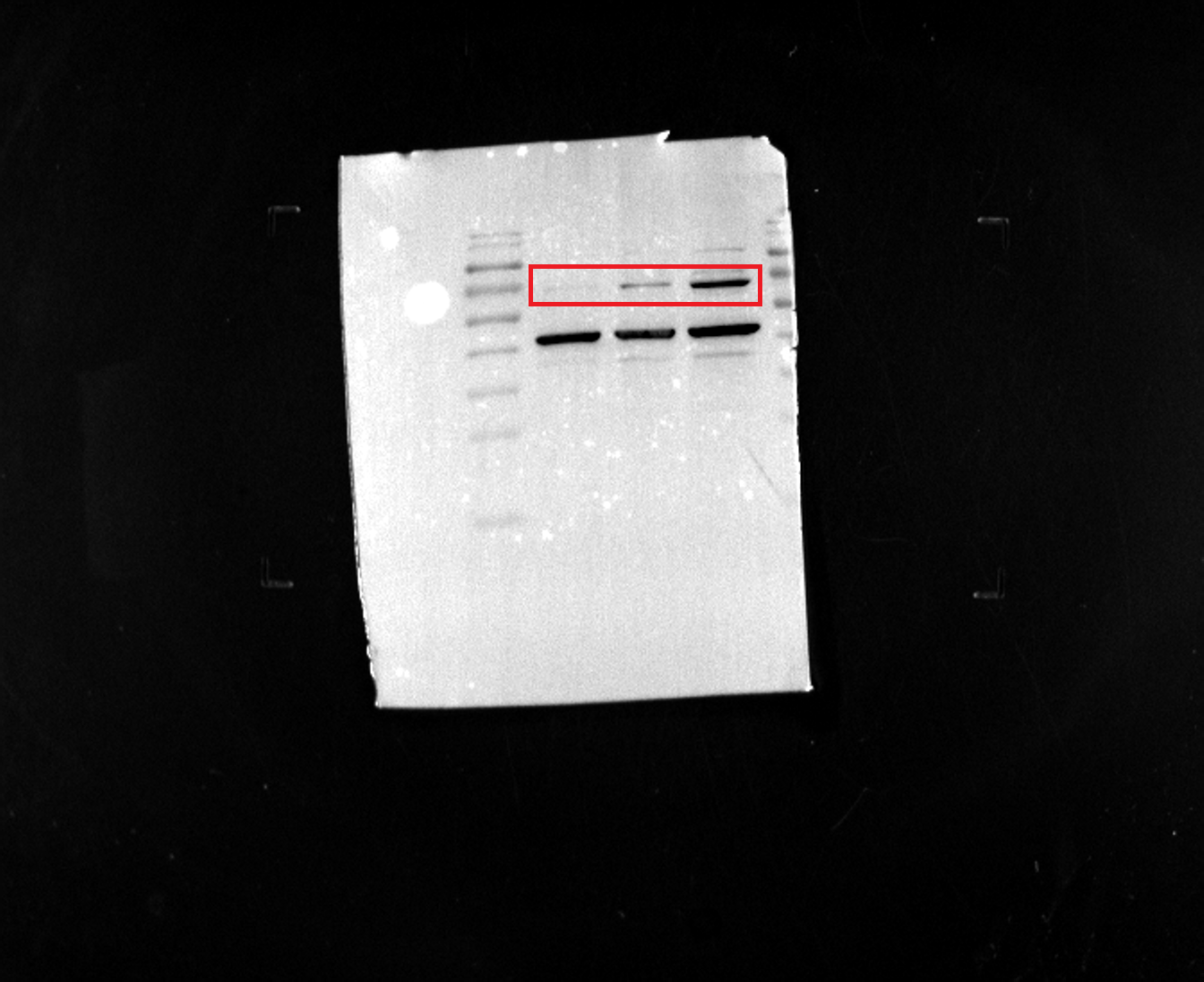


(4)TLR4


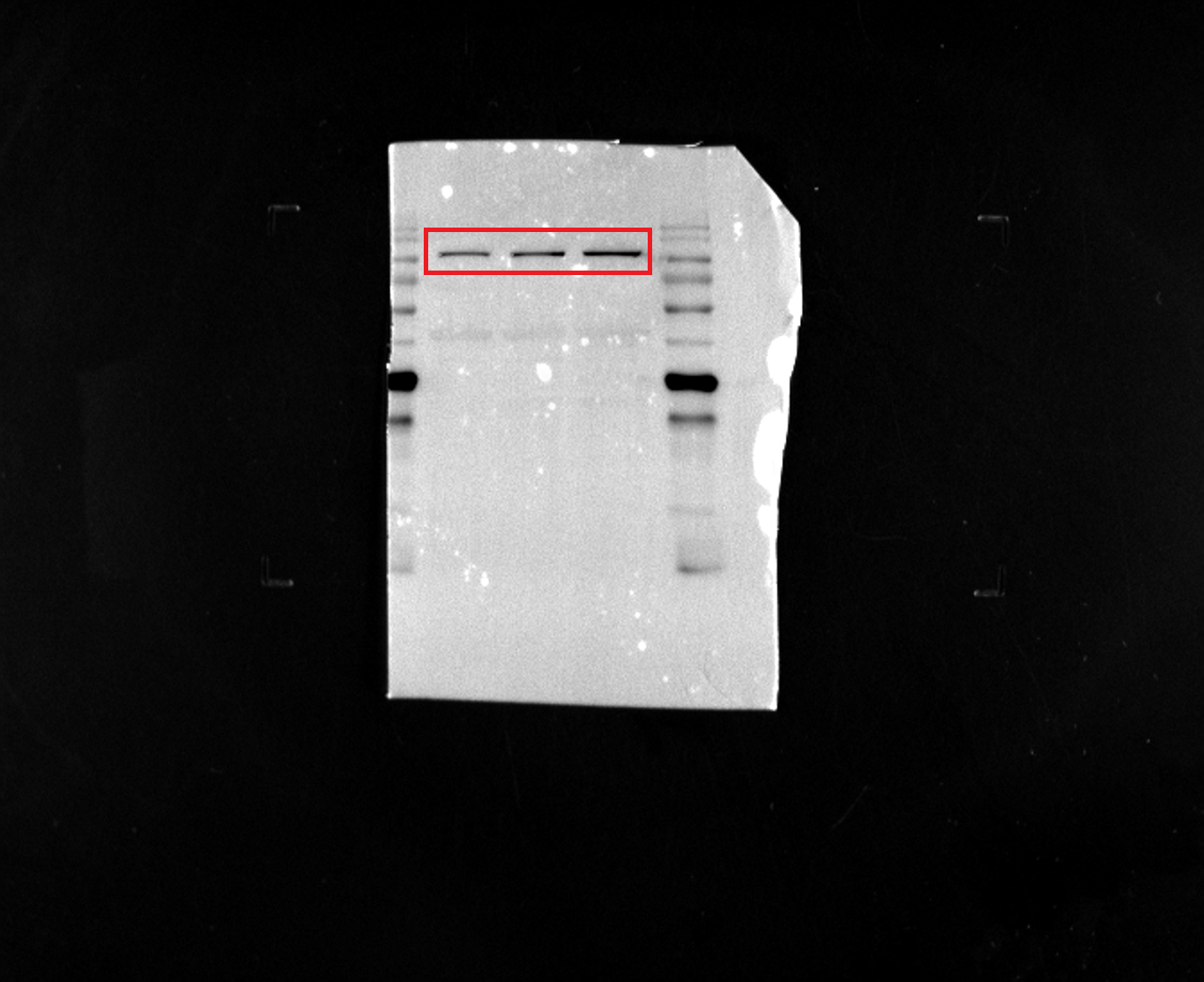


(5) NLRP6


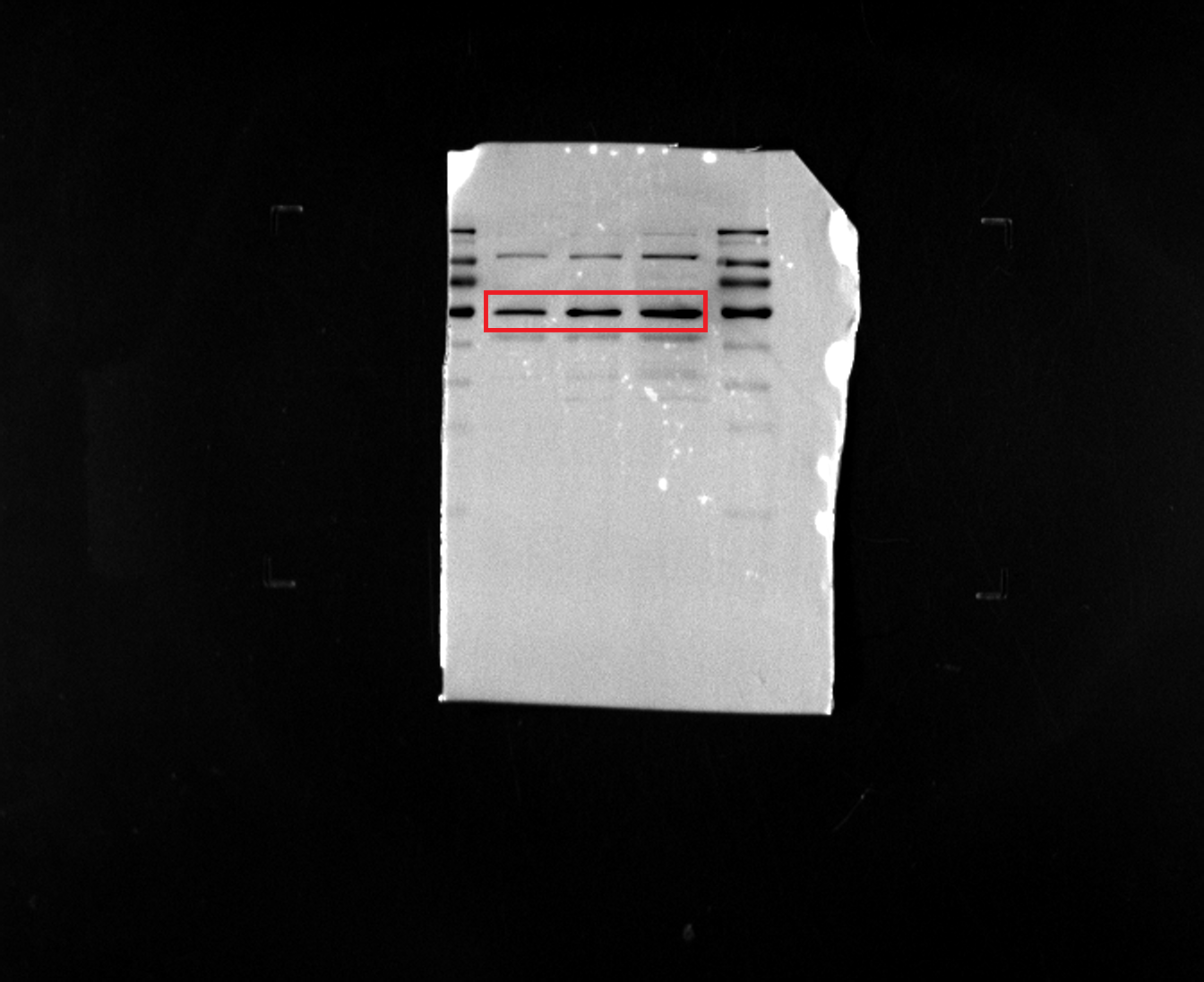

Supplement: Supplementary file 6 [file Table_6.DOCX]
