# Supplementary figures and images for: Multidrug-resistant mammary pathogenic Escherichia coli ST479 isolated from Holstein dairy cows in Jiangsu, China
Source: Front Microbiol. 2026 Mar 3;17:1737656. doi: 10.3389/fmicb.2026.1737656 (PMC13067290; doi:10.3389/fmicb.2026.1737656)

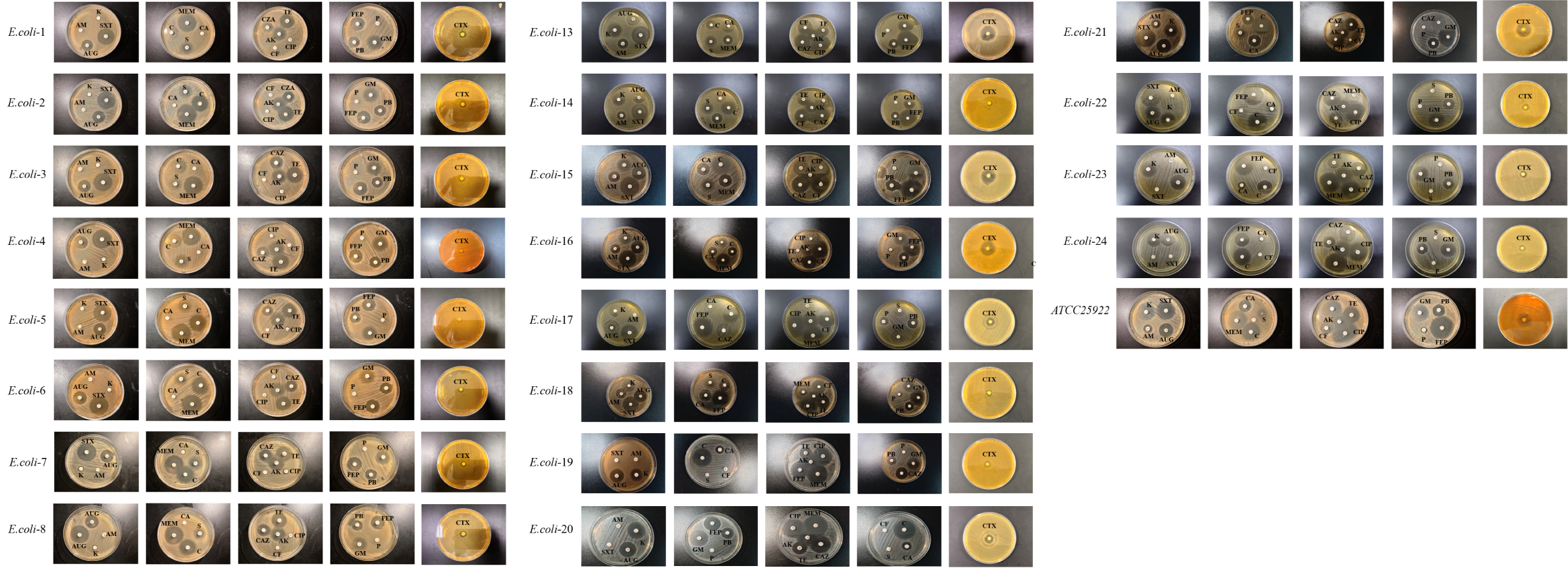

Supplement: Supplementary file 7 [file Table_7.DOCX]
